# Draft Genome Sequences for Seven *Streptococcus parauberis* Isolates from Wild Fish in the Chesapeake Bay

**DOI:** 10.1128/genomeA.00741-16

**Published:** 2016-08-18

**Authors:** Ashley Haines, Emily Nebergall, Elvira Besong, Kimaya Council, Onaysha Lambert, David Gauthier

**Affiliations:** a Department of Biology, Norfolk State University, Norfolk, Virginia, USA; b Department of Biological Sciences, Old Dominion University, Norfolk, Virginia, USA

## Abstract

*Streptococcus parauberis* is a pathogen of cattle and fish, closely related *Streptococcus uberis and Streptococcus iniae*. We report the genomes of seven *S. parauberis* strains recovered from striped bass (*Morone saxatilis*) in the Chesapeake Bay. The availability of these genomes will allow comparative genomic analysis of Chesapeake Bay *S. parauberis* strains versus *S. parauberis* cultured from other animal hosts and geographic regions.

## GENOME ANNOUNCEMENT

*Streptococcus parauberis* is a Gram-positive lactic acid bacterium first recognized as an agent of bovine mastitis in domestic cattle, where it was previously considered a subtype of *Streptococcus uberis* (1). *S. parauberis* is an emerging pathogen in the aquaculture industry and has caused epizootics of streptococcosis in multiple geographic locations, including Spain, in cultured turbot (*Scopthalmus maximus*) (2); South Korea, in olive flounder (*Paralichthys olivaceaus*) (3); and Japan, in olive flounder and in a cultured sea bass species *Sebastes ventricosus* (4, 5). Recently, *S. parauberis* was cultured from wild striped bass (*Morone saxatilis*) from the Chesapeake Bay, representing the first detection of this bacterial pathogen in fish in North America and its first detection in a wild host population (6). We report the genomes of seven *Streptococcus parauberis* strains cultured from striped bass hosts in the Chesapeake Bay.

Purified isolates were grown for 48 h in 5 mL of BHI broth at 30°C. Genomic DNA was isolated from purified isolates using DNEasy Blood and Tissue kits (Qiagen). Genomes were sequenced using Illumina MiSeq or HiSeq sequencing platforms at the University of Texas at Austin Genomic Sequencing and Analysis Facility. Then 125-bp paired-end reads were downsampled to either 50× or 100× coverage using FastqToCA and *de novo* assembled into contigs using Celera Assembler v 1.1, 6.1, 8.2, or 8.3 (7) with default parameters. Contigs were then annotated using Prokka v 1.11 (8).

A full description of these strains will be included in a future report with the results of comparative *S. parauberis* genomic analysis.

### Accession number(s).

These draft genomes have been deposited in GenBank under the accession numbers listed in Table 1. The versions described in this paper are the first versions.

**TABLE 1:** Characteristics of 7 *Streptococcus parauberis* isolates from striped bass (*Morone saxatilis*) in the Chesapeake Bay

Strain name	Accession no.	SRA*^*a*^* accession no.	Total contig length (bp)	No. of contigs	G+C content (%)	No. of CDSs*^*b*^*	No. of tRNAs
N11	LIXR00000000	SRR3231596	1,999,273	14	35.50%	2,019	23
N198_2	LHAC00000000	SRR3152169	1,975,829	14	35.50%	1,972	49
PL23	LHAD00000000	SRR3151460	2,020,482	12	35.50%	2,040	39
PL9	LJCT00000000	SRR3280351	2,012,234	23	33.90%	2,028	41
RP15	LRBI00000000	SRR3231612	2,012,630	12	39.00%	2,040	53
RP17	LRBJ00000000	SRR3231613	2,016,416	12	38.90%	2,049	53
RP25	LRBK00000000	SRR3231641	2,031,135	12	35.70%	2,057	57

a SRA, Sequence Read Archive.

b CDSs, coding sequences.
